# Supplementary material for: Reconciling Mining with the Conservation of Cave Biodiversity: A Quantitative Baseline to Help Establish Conservation Priorities
Source: PLoS One. 2016 Dec 20;11(12):e0168348. doi: 10.1371/journal.pone.0168348 (PMC5173368; doi:10.1371/journal.pone.0168348)
Supplement: S1 Dataset — (ZIP) [file pone.0168348.s002.zip › Taxa/Serra Norte/SN_2013/N5S/Lista N5S-42.pdf]

CAVIDADE N5S-0042

Classe	Ordem	Fam/Outros	Gên/Outros	Espécie	S-U	U-S	Zon.
Arachnida	Acari	Astigmata		jovem	X		E
Arachnida	Acari	Laelapidae	*Cosmolaelaps*	sp.1	X		E
Arachnida	Araneae	Caponiidae		jovem	X		P
Arachnida	Araneae	Corinnidae	*Creugas*	sp.1	1		E
Arachnida	Araneae	Ctenidae		jovem	1		P
Arachnida	Araneae	Ochyroceratidae	*Ochyrocera*	sp.1	X		P
Arachnida	Araneae	Oonopidae		jovem	X		E
Arachnida	Araneae	Pholcidae	*Leptopholcus*	sp.1		X	P
Arachnida	Araneae	Pholcidae	*Mesabolivar*	*aurantiacus*		X	E/P
Arachnida	Araneae	Pholcidae	*Mesabolivar*	sp.1	X		P
Arachnida	Araneae	Salticidae		sp.13	X	X	E
Arachnida	Araneae	Scytodidae	*Scytodes*	jovem	1		P
Arachnida	Araneae	Theridiidae		jovem	X		E
Arachnida	Araneae	Theridiosomatidae	*Plato*	sp.1		X	E/P
Arachnida	Opiliones	Cosmetidae	*Roquettea*	*singularis*		10	E/P
Arachnida	Pseudoscorpiones	Olpiidae		sp.1	2		E
Entognatha	Collembola	Paronellidae		sp.1	X	X	E
Entognatha	Collembola	Sminthuroidea		sp.2	X		P
Insecta	Blattodea	Polyphagidae		sp.1	2	1	P
Insecta	Coleoptera			jovem	X		P
Insecta	Diptera	Psychodidae	Phlebotominae	sp.		X	E/P
Insecta	Heteroptera	Dipsocoroidea		sp.	X		P
Insecta	Heteroptera	Reduviidae	Reduviinae	sp.	3	2	E/P
Insecta	Homoptera	Cixiidae		jovem	X	X	E
Insecta	Hymenoptera	Chalcidoidea		sp.2		X	E
Insecta	Hymenoptera	Formicidae	*Camponotus*	sp.2		X	P
Insecta	Hymenoptera	Formicidae	*Dolichoderus*	aff. *bispinosus*	X		E
Insecta	Hymenoptera	Formicidae	*Eurhopalothrix*	sp.1		X	E
Insecta	Hymenoptera	Formicidae	Myrmicinae	sp.1	X	X	E/P
Insecta	Hymenoptera	Formicidae	*Pheidole*	sp.2	X		E
Insecta	Hymenoptera	Formicidae	*Solenopsis*	sp.1		X	E
Insecta	Isoptera	Termitidae	*Nasutitermes*	sp.	X	X	E/P
Insecta	Isoptera	Termitidae	*Subulitermes*	sp.		X	P
Insecta	Lepidoptera	Noctuoidea		sp.1		1	E
Insecta	Lepidoptera	Noctuoidea		sp.5		1	E
Insecta	Lepidoptera			jovem	X		P
Insecta	Neuroptera	Myrmeleontidae		jovem	X	X	E
Insecta	Odonata	Zygoptera		sp.1	1		E
Insecta	Orthoptera	Phalangopsidae	*Paraclodes*	sp.		2	E
Insecta	Orthoptera	Phalangopsidae	*Phalangopsis*	sp.	6	5	P
Insecta	Orthoptera	Tettigoniidae	Listroscelidinae	sp.	1		P
Insecta	Psocoptera	Archipsocidae	*Archipsocus*	sp.1	X		E
Insecta	Psocoptera	Psyllipsocidae		jovem	X	X	E/P
Insecta	Thysanura	Nicoletiidae		jovem	X		P
Amphibia	Anura	Strabomantidae	*Pristimantis*	cf. *fenestratus*	2	1	P
